# The ability of different imputation methods for missing values in mental measurement questionnaires

**DOI:** 10.1186/s12874-020-00932-0

**Published:** 2020-02-27

**Authors:** Xueying Xu, Leizhen Xia, Qimeng Zhang, Shaoning Wu, Mingcheng Wu, Hongbo Liu

**Affiliations:** Public Health of School, China Medical University, No.77 Puhe Road, 110122 Shenyang, People’s Republic of China

**Keywords:** Imputation methods, Mental measurement questionnaires, Hot-deck imputation, Multiple imputation

## Abstract

**Background:**

Incomplete data are of particular important influence in mental measurement questionnaires. Most experts, however, mostly focus on clinical trials and cohort studies and generally pay less attention to this deficiency. We aim is to compare the accuracy of four common methods for handling items missing from different psychology questionnaires according to the items non-response rates.

**Method:**

All data were drawn from the previous studies including the self-acceptance scale (SAQ), the activities of daily living scale (ADL) and self-esteem scale (RSES). SAQ and ADL dataset, simulation group, were used to compare and assess the ability of four imputation methods which are direct deletion, mode imputation, Hot-deck (HD) imputation and multiple imputation (MI) by absolute deviation, the root mean square error and average relative error in missing proportions of 5, 10, 15 and 20%. RSES dataset, validation group, was used to test the application of imputation methods. All analyses were finished by SAS 9.4.

**Results:**

The biases obtained by MI are the smallest under various missing proportions. HD imputation approach performed the lowest absolute deviation of standard deviation values. But they got the similar results and the performances of them are obviously better than direct deletion and mode imputation. In a real world situation, the respondents’ average score in complete data set was 28.22 ± 4.63, which are not much different from imputed datasets. The direction of the influence of the five factors on self-esteem was consistent, although there were some differences in the size and range of OR values in logistic regression model.

**Conclusion:**

MI shows the best performance while it demands slightly more data analytic capacity and skills of programming. And HD could be considered to impute missing values in psychological investigation when MI cannot be performed due to limited circumstances.

## Background

Mental health is mostly measured by one or more psychometric scales that commonly include a set of functional items. It is inevitable to lead to missing items in psychological measurement, in the process of completing scales, due to the existence of many factors [1]. Any missing item will result in the loss of the whole scale score because items are collected to calculate subscale scores and total scale scores. Therefore, incomplete data are of particular important influence in mental measurement questionnaires (psychologic instruments). While researchers generally pay less attention to this deficiency. Many studies fail to mention the existence of missing and the methods of handing it [2, 3]. Others merely alluded to the default method or typically discard of samples with missing value to obtain a complete dataset [4]. These methods, known as listwise deletion or complete case analysis, reduce even more sample size available for analysis [5]. It results in reducing the statistical power (by reduced sample size) and causing greater selection biases if observations with missing values are only excluded from the analysis [6]. In recent years, many statistical methods have been proposed to impute missing values. The most common method consists in imputing a missing value by the average response of the other items or mean imputation in total scores of scale. Such a method is clearly recommended in scoring manuals of widely used quality of life questionnaires such as SF-36 and QLQ-C 30 [7, 8] though it might be inadequate especially when the rate of missing data is high [9–11].

Incomplete items of psychometric scales are a commonly encountered scenario in cross-sectional observational studies. Most experts, however, mostly focus on clinical trials and cohort studies or longitudinal studies during more than 20 years of research on missing values. There are few studies on the missing values in psychological tests in cross-sectional studies. Although a few articles cover this topic, they are limited to one aspect. For example, Shrive et al. has compared imputation techniques only based on the Zung depression scale, only considered one simulation (one distribution of the missing data) for each scenario and used a dichotomous criterion (“diseased” or “not diseased” according to the score) for comparisons. It did not make comparisons between the scale scores [11]. These results were hardly to reasonably extending to other questionnaires constructed according to classical test theory.

Therefore, based on the data from three cross-sectional surveys, we explored the actual effects of different missing rates from different populations and different scales considering the real-world evidence from the practical application point. Our aim to compare the accuracy and precision of four common methods for handling items missing from different psychology questionnaires according to the types of missingness and the item non-response rates, in order to provide advice to scientific researchers on the choice of appropriate imputation methods in their future work.

## Method

### Data sources

Considering the wider population and applicability of the scale, three common scales were selected from different age groups as the simulated imputation datasets, which are the self-acceptance scale (SAQ) of college students, the activities of daily living scale (ADL) of elderly people and self-esteem scale (RSES) of middle school students [12–14]. In enrolled dataset, samples with missing values are typically discarded to obtain a complete dataset. The SAQ dataset included 742 individuals with complete age, gender, the characteristics of parents, etc. The ADL dataset included 1242 elders with age, gender, the characteristics of daily living, etc. There are 3513 middle school students in RSES dataset also with complete age, gender, the characteristics of parents, etc. SAQ and ADL dataset, simulation group, were used to compare and assess the ability of different imputation methods. RSES dataset, validation group, was used to test the application of imputation methods. All datasets were complete on all required variables.

### Simulation of missing data

The mechanism of the missingness is important when imputing missing values. Missing item scores can be categorized into three types by Little and Rubin: when the missing data is independent to the actual or potential study variables, the losses are thought to be missing completely at random (MCAR) [15]. If the missingness due to issues related to the biological, psychological, social and/or cultural diversity of subjects, or depends on known or observed covariates, the non-issuance of the response is due to random causes (MAR). And the item nonresponse is classified as missing not at random (MNAR) if the probability of an item being missing depends on the true answer [16, 17]. In real world data, there is no way to verify that the data is MAR or MNAR though MCAR can be confirmed by Litter’s MCAR. Therefore, it is difficult to determine the missing data mechanism. Most scholars suppose that the missing of questionnaire data is at MAR, which use the relationships with other variables. In addition, most current imputation methods assume MAR in order to avoiding biased results. So the explanatory variables were assigned to missing under a MAR missing data mechanism in this study.

### Imputation methods

Four imputation methods are considered in this study. Among them, (1) the direct deletion method is to delete all subjects with missing values and conduct statistical analysis based on a complete dataset. It is the most common and simplest approach which was used in statistical software. (2) Mode imputation is one of the most naive and easiest methods for imputing missing values for categorical variables. The mode of the non-missing values of each variable was used to impute the missing values. (3) Hot-deck (HD) imputation refers to selecting the corresponding variable value of the observation most “similar” to the missing observation as the filling value of the missing observation. Generally, it is divided into two methods: sequential hot platform filling method and random hot platform filling method [18]. The most “similar” observation in sequential hot platform filling is selected in some order in the filling class. Random hot platform filling is randomly selected from the filling class. This research selects random Hot-deck imputation. (4) Multiple imputation (MI), which aims to produce a range of values that “approximate” the missing response [19]. MI uses a set of external covariates to generate a range of plausible values for each missing value (based on correlations between the covariates and the item to be replaced). The algorithm works by iteratively imputing the missing values based on the fitted conditional models until a stopping criterion is satisfied.

### Performance evaluation of imputation algorithms

Comparison of different imputation methods is performed as follows:

1. Absolute deviation. It is the absolute value of the difference of results between two data points of complete dataset and imputation dataset.
2. The root mean square error (RMSE) [20].
  $$ RMSE=\sqrt{\frac{\sum \limits_{i=1}^n{\left({y}_{ij}-{y}_{i0}\right)}^2}{n},} $$

where n = the number of simulated imputation in each missingness proportion,

*y*_*ij*_ *= statistics of ith imputation using imputation method j in each missingness proportion,*

*y*_*i0*_ *= statistics of ith imputation using complete dataset in each missingness proportion.*

Higher RMSE indicates larger differences between datasets imputed with the test methods. A narrower range of RMSE values indicates more stability in imputation method. Likewise, a wider range of RMSE values for each combination indicates less stability and therefore reliability in imputation method.

(3) Average relative error.
  $$ \mathrm{average}\ \mathrm{relative}\ \mathrm{error}=\frac{\sum \limits_{i=1}^n\left(\frac{y_{i0}-{y}_{ij}}{y_{i0}}\right)}{n}, $$

where n = the number of simulated imputation in each missingness proportion,

*y*_*ij*_ *= statistics of ith imputation using imputation method j in each missingness proportion,*

*y*_*i0*_ *= statistics of ith imputation using complete dataset in each missingness proportion.*

The vertical axis plots the percentage relative error for continuous variables and percentage misclassification error for categorical variables, while the horizontal axis groups the results according to the proportion of missing values. Each boxplot represents the error measure over 50 random replications.

### Statistical analysis

SAQ and ADL dataset were used as simulation groups. The missing rates in all datasets were set at 5, 10, 15, and 20% under a MAR missing data mechanism, respectively. And We repeated 50 times to simulate a MAR missing data and fill the missing values at each missing rate by four imputation methods before absolute deviation and RMSE of mean, standard deviation, correlation coefficient were calculated. If the results of all methods were similar, average relative error of these statistics will be continued to calculate or they will be computed except those imputation methods with less effective than others obviously in order to determine the preferred methods. RSES dataset, validation group, was analyzed the performance of the extensionality in a supposed real world situation by simulation different nonresponse rates one time. All analyses were finished by SAS 9.4.

## Result

### Results in simulated situations

The results of the comparisons of imputation methods for the analysis the absolute deviation of mean, standard deviation and correlation coefficient are shown in Table 1. The mean biases obtained by direct deletion are the biggest (0.583, 1.080, 1.453, and 1.586 in SAQ) and mode imputation is the most unstable under various missing proportions. MI has the best result, while the HD is not much different from it. HD imputation performed the lowest absolute deviation of standard deviation for each condition. And the performances of them are obviously better than direct deletion and mode imputation, especially in the high percentage of missing values of both scales.

**Table 1:** The absolute deviation for four imputation methods

Missingness proportion	Imputation methods	Absolute deviation for SAQ			Absolute deviation for ADL		
		Mean (SD)	SD (SD)	Correlation coefficient (SD)	Mean (SD)	SD (SD)	Correlation coefficient (SD)
5%	Direct deletion	0.583 (0.195)	0.153 (0.123)	0.028 (0.014)	0.218 (0.161)	0.144 (0.115)	0.022 (0.010)
	Mode	0.034 (0.021)	0.230 (0.026)	0.004 (0.002)	0.492 (0.026)	0.425 (0.039)	0.002 (0.001)
	HD	0.020 (0.015)	0.025 (0.019)	0.004 (0.002)	0.016 (0.011)	0.011 (0.008)	0.001 (0.001)
	MI	0.019 (0.013)	0.028 (0.020)	0.003 (0.002)	0.012 (0.010)	0.019 (0.014)	0.001 (0.001)
10%	Direct deletion	1.080 (0.324)	0.226 (0.176)	0.050 (0.024)	0.417 (0.299)	0.294 (0.214)	0.041 (0.017)
	Mode	0.065 (0.034)	0.463 (0.038)	0.006 (0.003)	0.999 (0.036)	0.856 (0.052)	0.004 (0.001)
	HD	0.032 (0.024)	0.050 (0.035)	0.006 (0.003)	0.045 (0.021)	0.019 (0.014)	0.002 (0.001)
	MI	0.028 (0.022)	0.070 (0.055)	0.006 (0.003)	0.020 (0.013)	0.030 (0.024)	0.002 (0.001)
15%	Direct deletion	1.453 (0.506)	0.353 (0.285)	0.074 (0.034)	0.721 (0.532)	0.507 (0.339)	0.059 (0.026)
	Mode	0.101 (0.060)	0.697 (0.044)	0.008 (0.004)	1.511 (0.044)	1.290 (0.060)	0.005 (0.002)
	HD	0.041 (0.030)	0.091 (0.042)	0.009 (0.004)	0.106 (0.027)	0.028 (0.022)	0.003 (0.001)
	MI	0.036 (0.033)	0.151 (0.202)	0.008 (0.003)	0.023 (0.016)	0.049 (0.036)	0.003 (0.002)
20%	Direct deletion	1.586 (0.690)	0.436 (0.325)	0.097 (0.046)	0.972 (0.658)	0.751 (0.525)	0.080 (0.034)
	Mode	0.141 (0.084)	0.925 (0.047)	0.009 (0.004)	2.019 (0.047)	1.717 (0.059)	0.007 (0.002)
	HD	0.050 (0.033)	0.161 (0.057)	0.012 (0.005)	0.182 (0.036)	0.024 (0.019)	0.004 (0.002)
	MI	0.048 (0.031)	0.287 (0.253)	0.010 (0.004)	0.024 (0.018)	0.067 (0.044)	0.005 (0.002)

*HD* Hot-deck imputation, *MI* nmultiple imputation, *SD* standard deviation

**Fig. 1:**
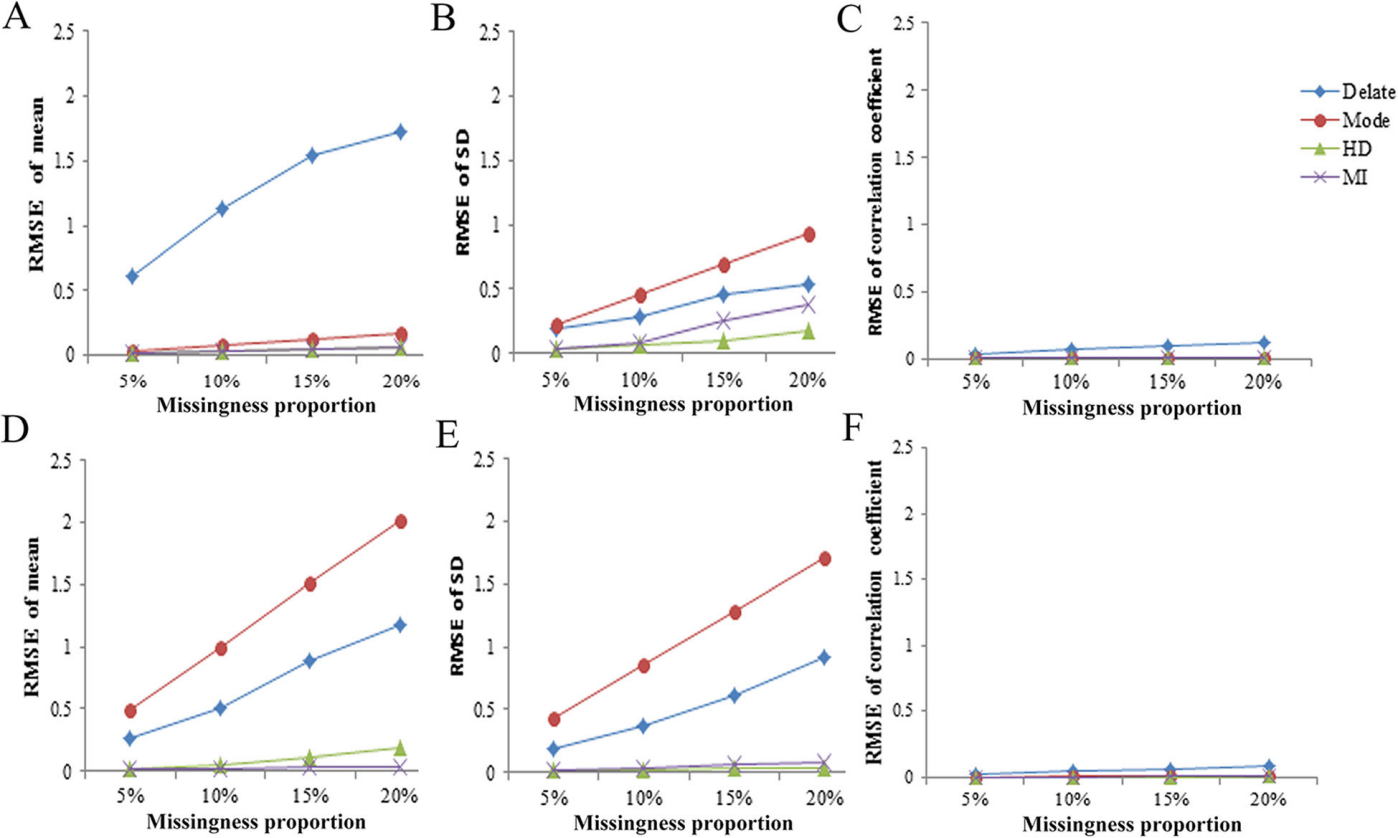
RMSE values for the 4 imputation methods. **a** - **c**. RMSE values for SAQ; **d** – **f**. RMSE values for ADL; RMSE, root mean square error; SAQ, self-acceptance scale; ADL, activities of daily living scale. SD, standard deviation

As shown in Fig. 1, the RMSE of mean values tended to increase with higher missingness rates under the same imputation approach. The RMSEs of mean and standard deviation in direct deletion and mode imputation of both two scales are higher obviously than other methods. In addition, except for the direct deletion technique, the differences between correlation coefficient calculated with imputed values and those “original values” were very small for other three methods in all the simulation scenarios. Next, direct deletion will be excluded in calculating the average relative error considering the less effective obviously in absolute deviation and RMSE of almost all statistics.

**Fig. 2:**
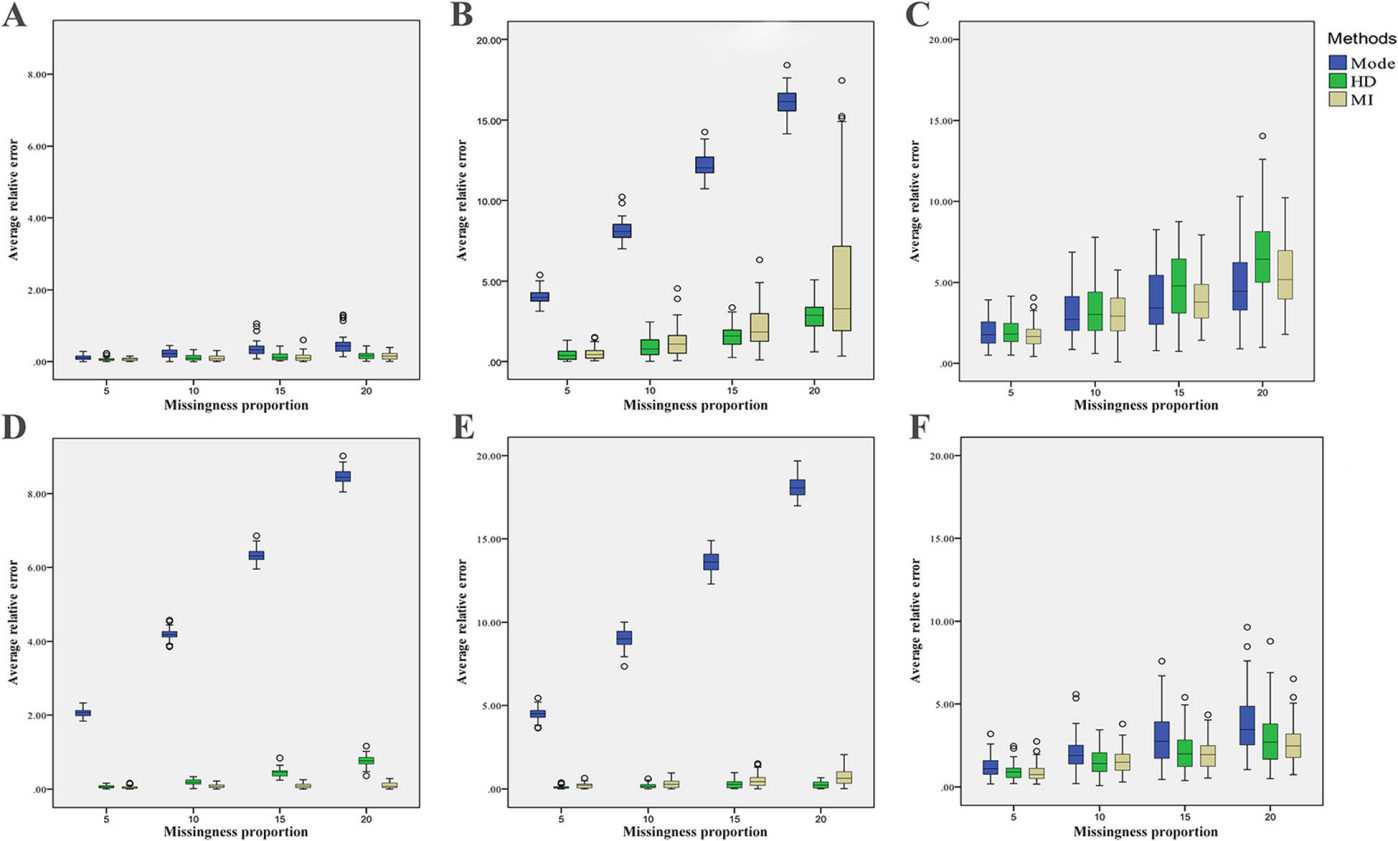
average relative error for the 3 imputation methods. **a**. average relative error of mean for SAQ. **b**. average relative error of SD for SAQ. **c**. average relative error of correlation coefficient for SAQ. **d**. average relative error of mean for ADL. **e**. average relative error of SD for ADL. **f**. average relative error of correlation coefficient for ADL

In ADL, the average relative error of mean and standard deviation by mode imputation are obviously greater than HD and MI methods. While in SAQ, the different of mean between three methods are not significant, but the average relative error of standard deviation by mode imputation is also bigger. The average relative error of mean in HD and average relative error of standard deviation in MI are slightly larger but not significant in both two scales. For correlation coefficient, the results of three imputation methods are similar (Fig. 2). In the end, we chose HD and MI to the next step because of their small deviation and stability.

### Results in real world situation

This portion evaluated the effect of HD and MI on the self-esteem scale in a real world situation. The complete data set included 3513 students from seventh grade to twentieth grade in the study using RSES. The average age was 16.3 years old and 1810 (51.5%) interviewees were male. 54.3% of adolescents were first-borns or only child (*n* = 1906) and the majority of them are from countryside (*n* = 3361). The parents of most students worked outside of their hometown for more than 5 years (father 56.0%, mother 52.2%).

**Fig. 3:**
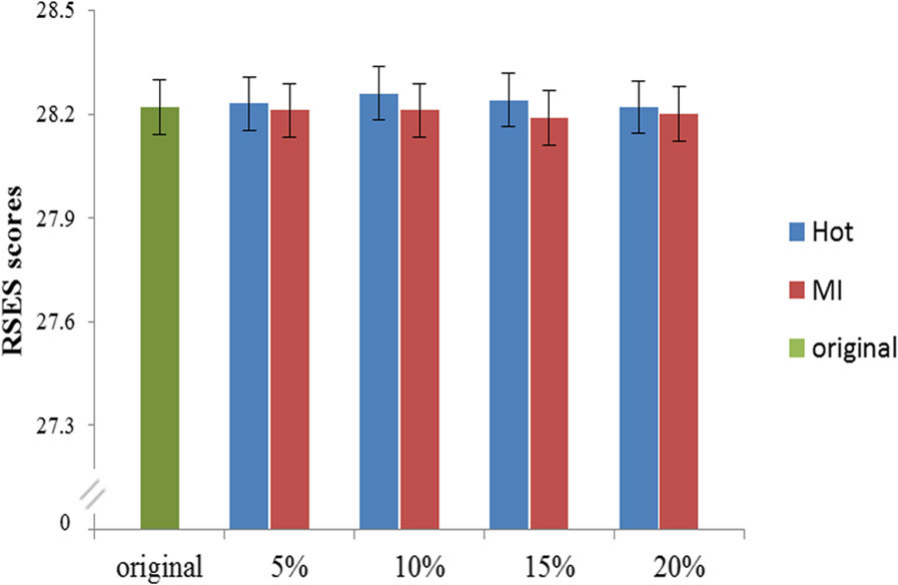
The average RSES scores of HD and MI

The average scores of RSES among middle school students in complete dataset, HD and MI under various missing proportions were showed in Fig. 3. The respondents’ average score in complete data set was 28.22 ± 4.63, which are not much different from imputed datasets even at the missingness proportion of 20%. The biggest deviation of score is 0.04 in HD imputation dataset of missingness rate of 10%, which is inconsistent with the previous result that the higher missing rates are the more errors get. It reveals that using HD and MI methods to interpolate missing values of scales has a good extension.

Based on the general analysis rules and methods of cross-sectional studies and mental health surveys, we further conduct descriptive study and hypothesis testing on the different characteristics of the RSES data. Compared with complete dataset, the two imputation methods also got the semblable result at different missing proportions (Table 2). The differences in all results are no more than 0.2 and the biggest deviation of score is 0.2 in HD imputation dataset of missingness rate of 20%. Overall, the average score of MI is more accurate than the result of HD imputation. However, we found that the variability of MI is significantly increased and the range of difference of standard deviation from the complete data is 0.06–3.44, which is far greater than the result of HD (0.01–0.29). Although the increase in variability did not affect the conclusion of comparison between different features in this study, this bias should be avoided as much as possible in actual work, which is due to the systematic error caused by MI method.

**Table 2:** The result of differences test of complete and impute data for RSES

variables		original		Missingness proportion of 5%				Missingness proportion of 10%				Missingness proportion of 15%				Missingness proportion of 20%			
				HD		MI		HD		MI		HD		MI		HD		MI	
		Mean (SD)	t / F value	Mean (SD)	t / F value	Mean (SD)	t / F value	Mean (SD)	t / F value	Mean (SD)	t / F value	Mean (SD)	t / F value	Mean (SD)	t / F value	Mean (SD)	t / F value	Mean (SD)	t / F value
Gender	Male	28.66 (4.57)	5.92	28.69 (4.55)	6.78	28.66 (5.51)	5.94	28.71 (4.50)	5.98	28.67 (5.48)	5.94	28.69 (4.48)	6.07	28.62 (5.44)	5.74	28.62 (4.34)	5.44	28.65 (5.39)	5.79
	Female	27.74 (4.64)		27.73 (4.63)		27.73 (5.53)		27.79 (4.63)		27.74 (5.49)		27.76 (4.59)		27.72 (5.43)		27.80 (4.52)		27.73 (5.34)	
Grade	Junior	28.30 (4.57)	1.46*	28.31 (4.56)	1.45*	28.29 (5.66)	1.07*	28.36 (4.52)	1.83*	28.30 (5.71)	1.06*	28.33 (4.48)	1.65*	28.27 (5.75)	1.01*	28.30 (4.42)	1.34*	28.28 (5.84)	1.01*
	Senior	28.06 (4.74)		28.07 (4.73)		28.04 (8.11)		28.07 (4.70)		28.05 (8.07)		28.06 (4.69)		28.03 (8.18)		28.08 (4.65)		28.04 (8.12)	
Record	Excellent	31.09 (4.74)	63.53	31.12 (4.77)	58.28	31.06 (5.89)	32.24	31.07 (4.79)	61.51	31.04 (5.92)	30.01	31.05 (4.76)	60.70	30.97 (5.90)	28.13	30.96 (4.80)	54.56	31.00 (5.93)	27.18
	Good	29.46 (4.46)		29.41 (4.46)		29.43 (5.62)		29.45 (4.41)		29.45 (5.58)		29.42 (4.46)		29.38 (5.62)		29.27 (4.35)		29.35 (5.54)	
	Average	28.13 (4.38)		28.14 (4.37)		28.12 (5.57)		28.18 (4.34)		28.12 (5.55)		28.13 (4.30)		28.08 (5.51)		28.15 (4.26)		28.10 (5.47)	
	Poor	26.46 (4.38)		26.53 (4.63)		26.49 (5.78)		26.56 (4.59)		26.53 (5.72)		26.58 (4.55)		26.55 (5.69)		26.66 (4.55)		26.59 (5.70)	
Residence	Urban	28.57 (4.86)	0.46*	28.58 (4.98)	0.29*	28.59 (6.07)	0.64*	28.56 (4.91)	0.25*	28.54 (6.01)	0.41*	28.75 (4.86)	0.74*	28.54 (5.96)	0.51*	28.71 (4.77)	0.67*	28.65 (5.85)	0.50*
	Rural	28.21 (4.62)		28.22 (4.60)		28.20 (5.87)		28.25 (4.57)		28.21 (5.83)		28.22 (4.54)		28.18 (5.78)		28.21 (4.49)		28.19 (5.72)	
Communication	Usually	28.87 (4.55)	32.29	28.86 (4.55)	29.15	28.86 (5.71)	8.63	28.90 (4.49)	31.29	28.87 (5.68)	8.11	28.86 (4.47)	30.47	28.83 (5.64)	8.18	28.85 (4.42)	30.57	28.86 (5.57)	8.15
	Sometimes	27.83 (4.34)		27.85 (4.33)		27.82 (5.49)		27.90 (4.35)		27.83 (5.50)		27.87 (4.31)		27.80 (5.44)		27.85 (4.26)		27.80 (5.39)	
	Scarcely	26.23 (5.23)		26.33 (5.18)		26.28 (6.42)		26.31 (5.04)		26.26 (6.27)		26.39 (5.10)		26.26 (6.30)		26.38 (4.94)		26.31 (6.27)	
	Never	25.68 (4.75)		25.63 (4.84)		25.66 (6.20)		25.81 (4.78)		25.79 (6.11)		25.81 (4.63)		25.78 (5.96)		25.74 (4.67)		25.72 (5.99)	
	Unclear	26.45 (4.94)		26.45 (4.92)		26.45 (6.29)		26.45 (5.02)		26.44 (6.34)		26.26 (4.99)		26.31 (6.30)		26.55 (4.82)		26.35 (6.23)	

*HD* Hot-deck imputation, *MI* multiple imputation. *SD* standard deviation

* *p* > 0.05

Logistic regression model was adopted to explore the relationship between variables in most investigations [21, 22]. In order to better describe and verify the possible bias of the analysis of the imputed data, we also used logistic regression to analyse the relationship between self-esteem and other variables. Self-esteem is divided into two categories (RSES score < 30 or RSES score ≥ 30) and gender, grade, academic record, residence and communication with relatives taking care of you as the independent variable. The logistic regression model was constructed in the complete dataset and the imputed datasets, respectively. The analysis results are shown in Table 3. The direction of the influence of the five factors on self-esteem was consistent, although there were some differences in the range of OR values between the data sets of original and imputation. The OR value of MI dataset is more similar to complete dataset than HD, but the largest difference of OR value is only 0.03 in the missing rate of 5% of the HD dataset.

**Table 3:** Influence on self-esteem in complete and impute data by multiple logistic regression models

Missingness proportion	Original OR (95% CI)	HD OR (95% CI)	MI OR (95% CI)
Complete data			
Gender	0.754 (0.648–0.877)	–	–
Grade	1.067 (1.022–1.115)	–	–
Record	0.674 (0.619–0.733)	–	–
Residence	1.054 (0.955–1.164)^a^	–	–
Communication	0.656 (0.589–0.731)	–	–
5%			
Gender	–	0.727 (0.625–0.845)	0.739 (0.634–0.861)
Grade	–	1.060 (1.015–1.106)	1.067 (1.021–1.115)
Record	–	0.680 (0.626–0.740)	0.674 (0.617–0.736)
Residence	–	1.030 (0.931–1.140) ^a^	1.045 (0.943–1.157) ^a^
Communication	–	0.686 (0.617–0.762)	0.665 (0.594–0.745)
10%			
Gender	–	0.718 (0.618–0.834)	0.712 (0.611–0.830)
Grade	–	1.062 (1.018–1.109)	1.059 (1.014–1.107)
Record	–	0.700 (0.645–0.760)	0.684 (0.627–0.746)
Residence	–	1.048 (0.950–1.157)^a^	1.052 (0.940–1.177)^a^
Communication	–	0.670 (0.603–0.745)	0.675 (0.604–0.754)
15%			
Gender	–	0.743 (0.639–0.864)	0.746 (0.639–0.871)
Grade	–	1.067 (1.022–1.114)	1.059 (1.010–1.110)
Record	–	0.691 (0.636–0.751)	0.696 (0.639–0.759)
Residence	–	1.082 (0.982–1.191)^a^	1.038 (0.929–1.159)^a^
Communication	–	0.708 (0.638–0.786)	0.683 (0.599–0.779)
20%			
Gender	–	0.748 (0.642–0.870)	0.726 (0.619–0.853)
Grade	–	1.074 (1.029–1.122)	1.053 (1.006–1.102)
Record	–	0.698 (0.642–0.759)	0.710 (0.645–0.781)
Residence	–	1.075 (0.975–1.185)^a^	1.057 (0.914–1.221)^a^
Communication	–	0.710 (0.639–0.789)	0.692 (0.618–0.774)

*HD* Hot-deck imputation, *MI* multiple imputation

^a^OR was not statistically significant

## Discussion

In this study, an extensive simulation study was performed in three psychology scales to compare the performance of four well-known methods of missing value imputation for missing data at random. To answer the question of whether to impute and to reflect the wide applicability of imputation methods in psychological research, we offer important points of the percentage of missing values and different scales that are mostly used to different populations. As expected, the imputation error of all statistics increases as the proportion of missing values increases in all approaches though the variation tends to reduce slightly, which is due to averaging over much more missing observations. Because the questionnaire survey is different from other studies of which results are independent, missing items would affect subscale and total scores. Even if there are not many missing values for independent items, it will lead to a larger proportion of respondents missing since the missing values of the items are distributed among different participants. There are, for example, only 564 individuals with the complete total scale scores in 1001 samples, when the percentage of missing items set to 20% in the dataset. Therefore, we recommend effective impute missing values to reduce the bias of missing on the questionnaire result.

In addition, the choice of imputation method does seem to drastically affect our results no matter in which psychological questionnaire. Both direct deletion and mode-imputation methods are simple to implement and have low computational requirements, but the performances of them are not satisfactory compared with other methods regardless of the different missing proportions or scales. This finding is in line with the conclusion from the literatures [23]. If the missingness is not related with the outcome, a direct deletion analysis will provide unbiased estimates [24]. Due to the particularity of the questionnaire, it may result in biased calculations in describing the distribution of test results or inferring relevant factors. It may, however, be reasonable to use mode-imputation analyses when computing correlation coefficient because the risk of bias is low and the precision could be bracketed with HD and MI. Therefore, we used both HD and MI to verify the accuracy and extensionality of the imputation method comparing with complete dataset when assumed real-world conditions.

The process of routine survey analysis was simulated in validation group. In general, the results of the MI and HD datasets are analogous to those of the original data. MI appeared to produce minimally biased estimates in describing the average and correlation analysis, but it can easily cause systematic errors in describing tendency of dispersion. The results of HD imputation are more stable no matter average level, hypothesis testing, or correlation analysis, which is similar to the results of the two questionnaires in the simulation dataset [25].

MI is a relatively sophisticated imputation method. It is mainly performed by the Markov Chain Monte Carlo (MCMC) procedure and requires a preliminary evaluation of missing data. The use of MI method demands more data analytic capacity and skills of programming. However, for a psychologist or clinician, it is difficult to implement it. Additionally, the biggest problem is that multiple data sets are generated in MI method, after that the statistics obtained by multiple imputation are the integration of many group statistics. So the display of some results and the use of analytical methods have significant limitations. MI may not display all the information of regular statistical analyses. For example, only the regression coefficients of each variable can be integrated and statistical inference can be given when multiple linear regression is performed with multiple imputation. While, there is no evaluation of the stability of the regression equation. And some statistical analysis methods, such as survival analysis, are currently difficult to multiple impute directly using software. Therefore, in practical work, MI imputation has limitations. In our study, the results of HD imputation are also similar to those of the complete dataset, especially in the variability of the data, which is more consistent than MI. It is not easy to produce false negative results due to less increased variability after data imputation by HD. In addition, the basic principle of the HD method is simple and a dataset is convenient to do statistical analyses after imputation. In this case, we recommend HD to fill the missing data because of its ease of implementation and the results can be accepted compared with MI in the psychological questionnaire.

## Conclusion

We would be wise to think about the missing-data problem in psychological scales prior to making decisions. MI shows the best performance while it demands slightly more data analytic capacity and skills of programming. And HD could be considered to impute missing values in psychological investigation when MI cannot be performed due to limited circumstances.

## Data Availability

The datasets used and/or analyzed during the current study are available from the corresponding author on reasonable request.

## Abbreviations

ADL: Activities of daily living scale
HD: Hot-deck imputation
MAR: Missing at random
MCAR: Missing completely at random
MI: Multiple imputation
MNAR: Missing not at random
RMSE: The root mean square error
RSES: Self-esteem scale
SAQ: Self-acceptance scale
